# Integrative analysis of metabolite and transcriptome reveals biosynthetic pathway and candidate genes for eupatilin and jaceosidin biosynthesis in *Artemisia argyi*

**DOI:** 10.3389/fpls.2023.1186023

**Published:** 2023-04-25

**Authors:** Suhyeon Lee, Hyo Jun Won, Seunghyun Ban, Yun Ji Park, Sang Min Kim, Hyoung Seok Kim, Jaeyoung Choi, Ho-Youn Kim, Jae Hoon Lee, Je Hyeong Jung

**Affiliations:** ^1^ Smart Farm Research Center, Korea Institute of Science and Technology (KIST), Gangneung, Gangwon, Republic of Korea; ^2^ Division of Bio-Medical Science & Technology, KIST School, University of Science and Technology (UST), Daejeon, Republic of Korea; ^3^ Department of Oriental Medicine Biotechnology, College of Life Sciences, Kyung Hee University, Yongin, Republic of Korea; ^4^ Department of Agricultural Biotechnology, Seoul National University, Seoul, Republic of Korea; ^5^ Research Institute of Agriculture and Life Sciences, Seoul National University, Seoul, Republic of Korea

**Keywords:** *Artemisia argyi*, flavonoid, eupatilin, jaceosidin, transcriptome, biosynthetic pathway

## Abstract

*Artemisia argyi* (*A. argyi*) is a medicinal plant belonging to the *Asteraceae* family and *Artemisia genus*. Flavonoids abundant in *A. argyi* are associated with anti-inflammatory, anticancer, and antioxidative effects. Eupatilin and jaceosidin are representative polymethoxy flavonoids with medicinal properties significant enough to warrant the development of drugs using their components. However, the biosynthetic pathways and related genes of these compounds have not been fully explored in *A. argyi.* This study comprehensively analyzed the transcriptome data and flavonoids contents from four different tissues of *A. argyi* (young leaves, old leaves, trichomes collected from stems, and stems without trichomes) for the first time. We obtained 41,398 unigenes through the *de-novo* assembly of transcriptome data and mined promising candidate genes involved in the biosynthesis of eupatilin and jaceosidin using differentially expressed genes, hierarchical clustering, phylogenetic tree, and weighted gene co-expression analysis. Our analysis led to the identification of a total of 7,265 DEGs, among which 153 genes were annotated as flavonoid-related genes. In particular, we were able to identify eight putative flavone-6-hydroxylase (*F6H*) genes, which were responsible for providing a methyl group acceptor into flavone basic skeleton. Furthermore, five *O*-methyltransferases (*OMT*s) gene were identified, which were required for the site-specific *O*-methylation during the biosynthesis of eupatilin and jaceosidin. Although further validation would be necessary, our findings pave the way for the modification and mass-production of pharmacologically important polymethoxy flavonoids through genetic engineering and synthetic biological approaches.

## 1 Introduction

*Artemisia argyi* (*A. argyi*) is a perennial medicinal plant that is widely distributed in Asia, Europe, and North America (Lee et al., 2009; Mei et al., 2016; Song et al., 2019). It belongs to the *Artemisia* genus of the *Asteraceae* family, which comprises over 500 species and subspecies (Bora and Sharma, 2011), many of which contain various and abundant bioactive components (Yoshikawa et al., 1996; Han et al., 2017; Lv et al., 2018; Cui et al., 2022). These plants are used in various fields, such as the herbal, food, cosmetic, and pharmaceutical industries, and therefore have high economic value (Ivanescu et al., 2021; Kshirsagar and Rao, 2021). A major contributor to the discovery of Artemisinin, which is isolated from *Artemisia annua*, and recognized for its antimalarial effect, was awarded the Nobel Prize in Physiology or Medicine in 2015 (Su and Miller, 2015). *A. argyi* has various pharmacological effects, including anti-inflammatory (Yun et al., 2016), anticancer (Seo et al., 2003), antioxidative (Lan et al., 2010), and antimicrobial activities (Wang et al., 2006). These effects are attributed to its secondary metabolites, including flavonoids (Han et al., 2017), organic acids (Han et al., 2017), and terpenoids (Yoshikawa et al., 1996). Flavonoids, especially the polymethoxy flavones eupatilin and jaceosidin, are abundant in the aerial parts, especially leaves of *A. argyi* (Lv et al., 2018; Cui et al., 2022). These compounds have been shown to induce apoptosis in human promyelocytic leukemia cells (Seo and Surh, 2001) and inhibit cervical cancer cells (Lee et al., 2005). Furthermore, Stillen^®^ (DA-9601, Dong-A ST Co., Seoul, Korea), a drug developed using eupatilin and jaceosidin has been approved and utilized as a phytomedicine for gastritis treatment (Seol et al., 2004).

Flavonoids are synthesized *via* the phenylpropanoid pathway, in which phenylalanine is converted into 4-coumaroyl-CoA (Falcone Ferreyra et al., 2012). Flavonoid biosynthesis begins with the condensation of three malonyl-CoA molecules with one 4-coumaroyl-CoA molecule by chalcone synthase (CHS). Naringenin chalcones can be further modified into various flavonoid subclasses, such as chalcones, flavones, flavonols, flavandiols, anthocyanins, and proanthocyanidins. The biosynthesis of flavones involves several steps. Chalcone isomerase (CHI) catalyzes stereospecific isomerization of naringenin chalcone to synthesize naringenin, resulting in the synthesis of eriodictyol by flavonoid 3*′*-hydroxylase (F3*′*H). The two flavanones, naringenin and eriodictyol, act as major substrates of flavone synthase (FNS) and are converted into the basic flavone skeletons, apigenin and luteolin, respectively. Although the downstream pathways of the polymethoxy flavones eupatilin and jaceosidin have not yet been clearly elucidated, they are known to be produced *via* a series of enzymatic reactions of hydroxylation and *O*-methylation. These reactions start from a flavone basic skeleton, such as apigenin, or luteolin. In plants, members of the cytochrome P450 monooxygenase (CYP450) and *O*-methyltransferases (OMT) families, catalyze these reactions, respectively (Tohge et al., 2018; Yonekura-Sakakibara et al., 2019).

Transcriptome sequencing coupled with integrated metabolite profiling is a promising strategy for the identification of biosynthetic genes of interest. Attempts have been made in *A. argyi* to identify the genes involved in terpenoid (Liu et al., 2018; Zhang et al., 2022; Chen et al., 2023) and flavonoid (Miao et al., 2022) biosynthesis. Variations in levels of gene expression and contents of target compounds among the sample groups allow comparative studies, thereby an appropriate sample selection plays a key role in the success of this approach. The aerial parts of *A. argyi* are covered with trichomes. Trichomes are epidermal outgrowths found on the surfaces of various plant organs, including leaves, stems, and reproductive structures (Schilmiller et al., 2008; Frerigmann et al., 2012; Xiao et al., 2016; Tissier et al., 2017). Trichomes can be divided into glandular and non-glandular trichomes based on their structure and function. Glandulartrichomes contain glandular cells that secrete and store various secondary metabolites, such as terpenoids, alkaloids, and flavonoids, which play important roles in plant defense against herbivores, pathogens, and abiotic stress. Non-glandular trichomes, on the other hand, are composed of one or more layers of elongated cells and provide physical protection against various environmental factors, such as UV radiation, desiccation, and insect damage. The preliminary tests conducted in our group indicated that eupatilin and jaceosidin were abundant in trichomes collected from the surface of stems, compared to trichome-removed stem tissues. This result was further supported by recent studies which showed significant variations in their contents depending on the types of trichomes on leaves of *A. argyi* (Cui et al., 2022; Miao et al., 2022). In this regard, we chose four different tissues based on the presence or absence of trichomes and differences in their densities, and explored the differences in levels of gene expression and contents of target compounds among the sample.

Overall, the biosynthesis of secondary metabolites in plants is a complex and dynamic process that is not yet fully understood. In this study, we aimed to shed new light on the biosynthesis pathways of eupatilin, jaceosidin, and their precursors in *A. argyi*, and to identify the key genes and enzymes involved in this process. Using a combination of liquid chromatography and RNA-seq analyses of different tissue types, we aimed to provide a comprehensive understanding of the biosynthetic pathways of these important secondary metabolites, which may have important implications for the development of novel therapeutics and industrial applications.

## 2 Materials and methods

### 2.1 Plant materials

*A. argyi* plants (accession number AA1801), were obtained from the National Institute of Horticultural and Herbal Science, Rural Development Administration, Republic of Korea, and propagated asexually *via* rhizomes. The propagules were grown in a growth chamber under 22°C/18°C (day/night), with a 16-h photoperiod, 200 μmol/m^2^/s light intensity, and 50% relative humidity for 8 weeks. For the flavonoid quantification and transcriptome analyses, we collected the 3rd to 5th and 15th to 17th leaves counted from the shoot apical meristem, which were designated as ‘young leaves’ and ‘old leaves’, respectively. After removing the 10th to 17th leaves along with their petioles, the remaining stem was immediately frozen using liquid nitrogen, and the trichomes attached to the epidermal layer were collected using a razor blade and designated as the ‘trichome’ sample. The remaining stem without trichomes was designated as the ‘stem’ sample. Histochemical analysis of the stem transverse section was conducted by light microscopy to ensure the successful removal of the trichomes attached to the stem surface (**Supplementary Figure S1**). Each sample was prepared from three biological replicates.

### 2.2 Microscopic analysis of trichomes

The overall morphological characteristics of young and old leaves were analyzed using a dissecting microscope system, consisting of a Leica M60 microscope and a Leica IC90E digital camera with LAS version 4.13 software (Leica Microsystems, Wetzlar, Germany). The adaxial and abaxial surfaces of young and old leaves, as well as the stem surface, were imaged using a scanning electron microscope (SEM) to analyze the distribution and density of trichomes. The samples were fixed with 2.5% glutaraldehyde, followed by 2% osmium tetroxide post-fixation, dehydration, and drying using a hexamethyldisilazane (HMDS) solvent. The samples were then examined by a field emission-scanning electron microscopy with FEI Teneo VolumeScope (FEI, Hillsborough, USA) at the Advanced Analysis Center, Korea Institute of Science and Technology (Seoul, Korea). The density of glandular trichomes was measured in triplicate from the same-sized images of the samples.

### 2.3 Liquid chromatography analysis of flavonoids

For the quantification of each flavonoid, samples from each tissue were freeze-dried with three biological replicates. 50 mg of a freeze-dried powder sample was mixed with 900 μL of methanol and 100 μL of quercetin (as an internal standard at 250 μg/mL in methanol) and then ultrasonic extraction was performed for 60 minutes at 30°C. Filtration was conducted using a 0.45 μm syringe filter, followed by HPLC analysis.

The HPLC analysis was performed using an Agilent (Palo Alto, CA) Series 1200 liquid chromatography system and a Phenomenex Gemini C18 column packed with 5 μm particles (4.6 x 250 mm) at 35°C column oven. Acetonitrile and water (containing formic acid 0.1%) were used as a mobile phase. The binary mobile phase consisted of Solvent A (0.1% formic acid in water) and Solvent B (0.1% formic acid in acetonitrile) and was applied with a gradient program under the following conditions: 0 minutes, 10% B; 60 minutes, 20% B; 65 minutes, 23% B (isocratic for 30 minutes at 23% B); 95 minutes, 23% B; 120 minutes, 30% B; 150 minutes, 65% B. The injection volume was 10 μL, and the flow rate was set at 1.0 mL/min. UV detection was performed at 330 nm for fingerprint analysis. Freshly prepared calibration standards for each flavonoid were analyzed, and the contents of each compound were determined by standard curve fitting.

### 2.4 Total RNA isolation, library construction, and RNA-seq

Total RNA was isolated from four different tissue samples, young leaves, old leaves, trichomes, and stem using TRIzol (Life Technologies, Carlsbad, USA) and RNeasy Plant RNA kit (Qiagen, Hilden, Germany), followed by DNase treatment and RNA integrity verification with Ambion DNA-free kit (Thermo Fisher Scientific, Vilnius, Lithuania) and Agilent 2100 Bioanalyzer (Agilent Technologies, Santa Clara, USA), respectively, according to the manufacturer’s instruction. For RNA-seq, 12 paired-end cDNA libraries, representing three biological replicates per four different tissue samples, were constructed using TrueSeq mRNA kit (Illumina, San Diego, USA), and high-throughput sequencing was performed using Illumina HiSeq X Ten (Illumina).

### 2.5 *De novo* assembly, read mapping, annotation, and differential expression analysis

The sequence reads were pre-processed with Cutadapt for trimming adapter and DynamicTrim and LengthSort from the SolexaQA package for trimming low-quality sequences (Cox et al., 2010; Martin, 2011). The cleaned reads were subjected to *de novo* assembly using the Trinity version 2.8.6, which recovers full-length transcripts and splicing isoforms based on the de Bruijn graph by single k-mer (Grabherr et al., 2011), and aligned to the assembled transcripts using Bowtie 2 version 2.1.0 (Langmead and Salzberg, 2012). In the Trinity program, multiple isoform (transcript) information corresponding to a single locus is obtained. Among these isoforms, we selected the representative sequence with a longer assembly length and higher expression level. For annotation, all unique transcripts were analyzed using InterProScan and BLASTX against the non-redundant NCBI database under the taxonomy restriction to Viridiplantae (NCBI-NR). DESeq package in R was used to normalize the number of reads and identify differentially expressed genes (DEGs) with thresholds of | log2(fold change) | ≥ 1 and an adjusted p-value (false discovery rate) < 0.01 (Anders and Huber, 2010). All candidate genes involved in phenylpropanoid and flavonoid biosynthesis were further identified by tBLASTn search against the NCBI-NR database. GO term enrichment analysis was conducted through the Blast2GO program (FDR p-value: 0.05), and KEGG enrichment analysis was performed using an in-house script.

### 2.6 Weighted gene co-expression network analysis

WGCNA was conducted with normalized read counts of the 7,265 DEGs using an R package (Zhang and Horvath, 2005; Langfelder and Horvath, 2008) with the following parameter. To ensure that the scale-free topology index is greater than 0.8, three biological replicates of four different samples were used separately. Topological overlap matrix (TOM)- signed, power β=14, minimal module size=30, reassign threshold=0, and branch merge cut height=0. Eigengene (the first principal component of a given module) value was calculated and used for association analysis between a module and measured metabolites from four different tissues.

Hierarchical clustering heatmap was generated by converting the normalized expression values into Z-scores using the formula Z=(Log2(normalized count+1)-mean)/standard deviation. For metabolites, Z-scores were calculated using the same formula, but the amount of metabolite was used instead of normalized count.

### 2.7 Phylogenetic tree analysis

The phylogeny was inferred by Maximum Likelihood method, Le_Gascuel_2008 mode, and JTT substitution model available in software MEGA X (Kumar et al., 2018).

### 2.8 Statistical analyses

Analysis of variance (ANOVA), Tukey’s honest significant difference (Tukey HSD) procedures, and unpaired t-test were conducted using R v4.2.1 (Team, 2023).

## 3 Results

### 3.1 Differences in trichomes density among different tissues of *A argyi*

**Figure 1:**
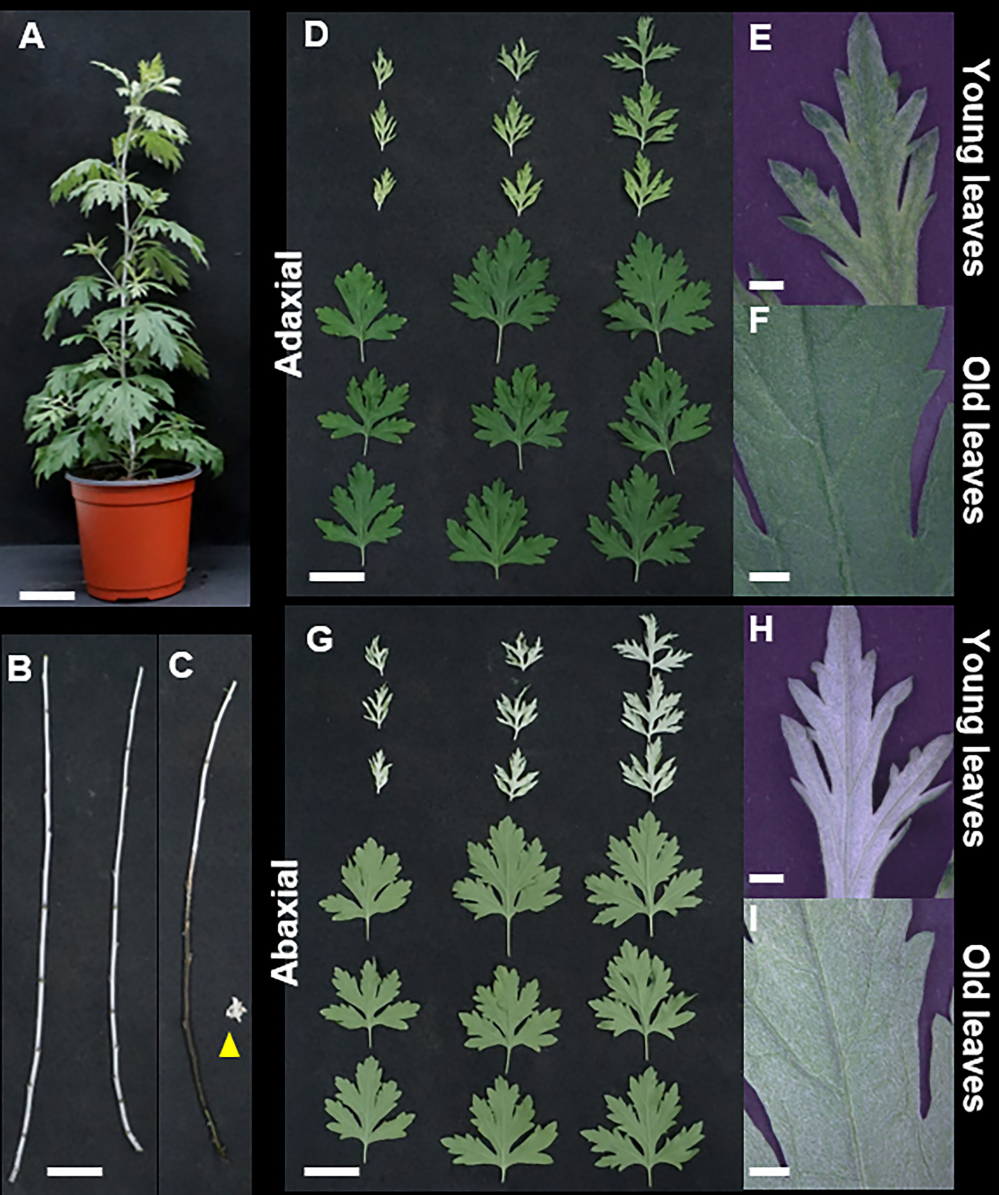
Morphological characteristics of *Artemisia argyi*. **(A)** Whole plant. **(B)** Stems. **(C)** The part where trichomes have been removed from the stem appears black, and the yellow triangle indicates the stripped trichome. **(D-F)**. Adaxial leaf surfaces in young and old leaves. **(G-I)** Abaxial surfaces in young and old leaves. Scale bars; **(A-D)**, G = 5 cm, **(E, F, H, I)** = 1 mm.

Visual and light microscopic analysis of the aerial parts of *A. argyi* revealed that the surfaces of the leaves and stems were covered with whitish trichomes. In **Figure 1**, it can be seen that the young leaves and abaxial surface display a brighter green color than the old leaves and the adaxial sides of both young and old leaves. The brightest color was observed on the abaxial surface of young leaves, indicating a higher density of trichomes. Trichome density was higher in developing young leaves and abaxial surfaces than in physiologically older leaves and adaxial surfaces.

**Figure 2:**
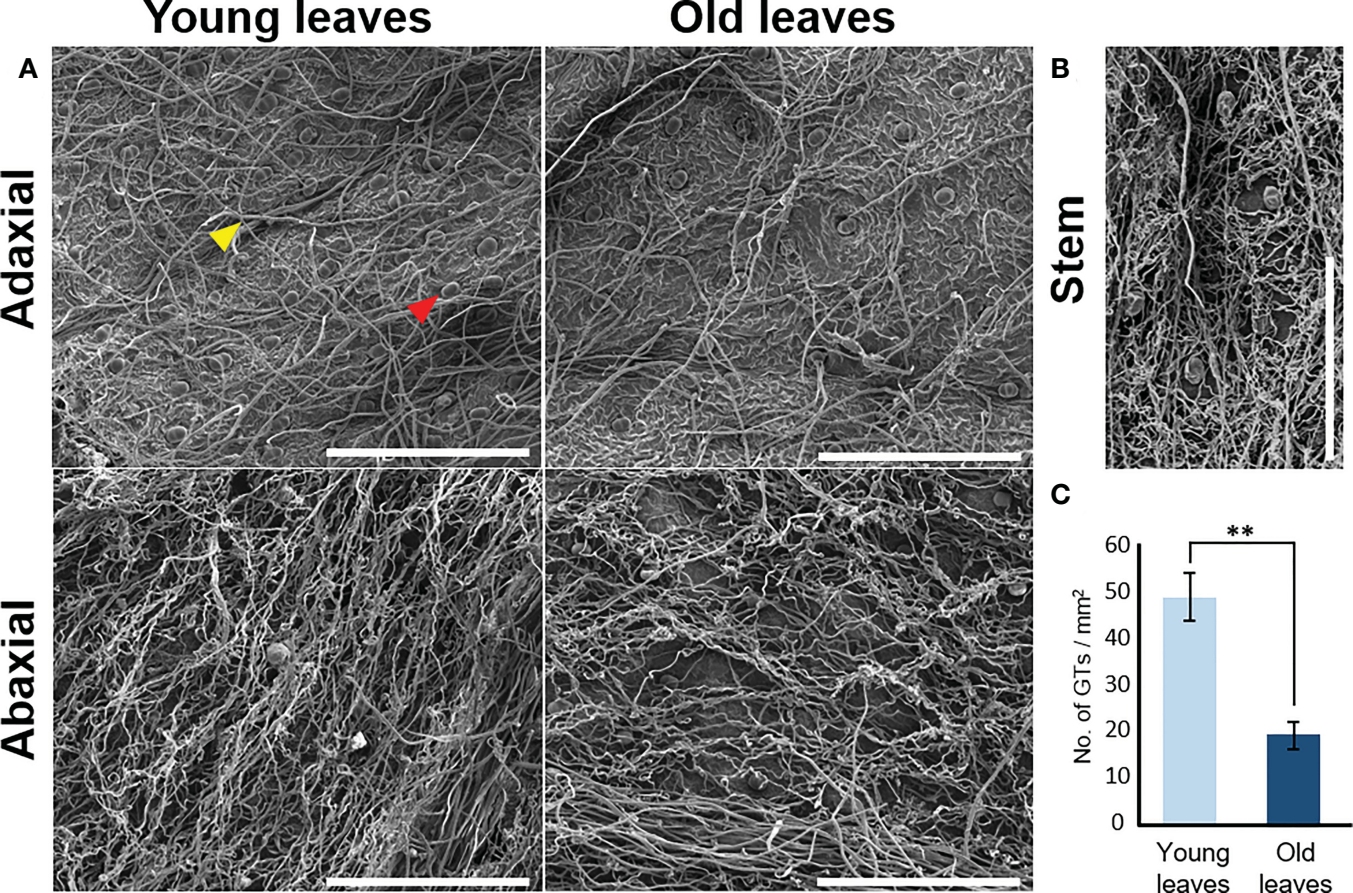
Micromorphology of trichomes of *Artemisia argyi*. **(A)** Distribution of trichomes on adaxial and abaxial surfaces of young and old leaves using a scanning electron microscope. Red triangle indicates glandular trichome and yellow triangle indicates non-glandular trichome. Scale bars: 500μm. **(B)** Distribution of trichomes in the stem. Scale bars: 500μm. **(C)** Glandular trichome (GT) density difference on adaxial surface between young and old leaves. Statistical differences were examined by two-tail method of unpaired Student’s t-test (*n*=3). Symbols denote statistical significance (** , <0.01).

High-resolution SEM was used to further analyze the morphology and density of the trichomes. The surfaces of the leaves and stems had both non-glandular and glandular trichomes (**Figures 2A, B**). Non-glandular trichomes were denser and curlier on the abaxial side than on the adaxial side of the leaves (**Figure 2A**). Elliptical ball-shaped glandular trichomes were present on both the abaxial and adaxial surfaces of the leaves. The density of glandular trichomes was found to be 2.6-fold higher in young leaves (49 ± 5.4/mm^2^) compared to old leaves (19 ± 2.9/mm^2^) on the adaxial surface (**Figure 2C**). However, the density of glandular trichomes on the abaxial surfaces of leaves and stems could not be quantified owing to the hidden glandular trichomes underneath multiple dense layers of non-glandular trichomes (**Figure 2A**).

### 3.2 Flavonoids quantification in different tissues of *A argyi*

**Figure 3:**
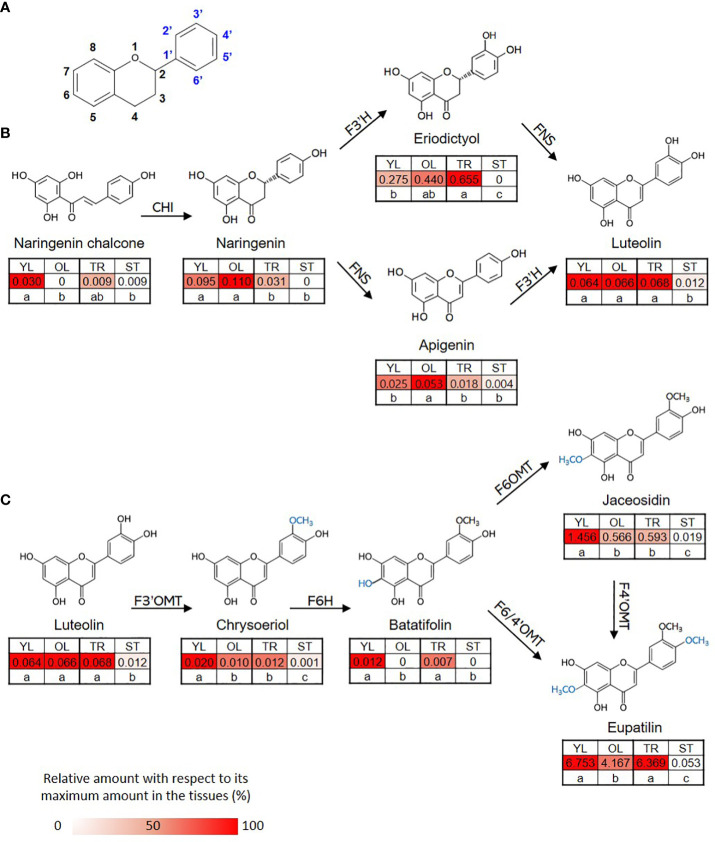
Proposed biosynthetic pathway for eupatilin and jaceosidin. **(A)** Basic structure of flavone. **(B)** Previously established biosynthetic pathway towards flavone basic skeletons, apigenin and luteolin. CHI; Chalcone-flavanone isomerase, FNS; Flavone synthase, F3´H; Flavanone-3´-hydroxylase. **(C)** Proposed remaining steps for eupatilin and jaceosidin biosynthesis starting from luteolin. F3’OMT; Flavonoid-3´-*O*-methyltransferase, F6H; Flavone-6-hydroxylase, F6OMT; Flavonoid-6-*O*-methyltransferase, F6/4’OMT; Flavonoid- 6/4´-*O*-methyltransferase. Hydroxylation and *O*-methylation by corresponding enzymes are shown in blue. YL represents young leaves, OL represents old leaves, TR represents trichomes, and ST represents trichome-removed stem. Numbers in the heatmap boxes represent the measured amount of the metabolite in mg/g dry weight of sample. The heatmap represents the relative abundance of the metabolite in each tissue as a percentage, relative to its maximum amount detected in any tissue. Different letters within rows indicate significant difference based on one-way ANOVA and Tukey’s HSD test (*n*=3, p < 0.05).

The content of eupatilin, jaceosidin, and their putative intermediates was determined in four different tissues selected based on the differences in trichome distribution and density to identify their biosynthetic genes showing a correlation between the gene expression levels and the metabolite contents (**Figure 3**). The flavonoid content in the known biosynthetic pathway from naringenin chalcone to luteolin varied among tissues, with stem samples showing the lowest content (**Figure 3B**). In a newly proposed pathway from luteolin to eupatilin and jaceosidin *via* chrysoeriol and batatifolin, the putative intermediates (chrysoeriol and batatifolin) and final products (eupatilin and jaceosidin) showed significantly higher concentrations in young leaves and trichomes than in old leaves and trichome removed stems, respectively (**Figure 3C**). All of the flavonoids involved in the pathway from luteolin to jaceosidin and eupatilin showed the lowest content in the stem where trichomes were completely removed (**Figure 3C**).

### 3.3 RNA-seq, *de novo* assembly, and annotation

To identify the genes responsible for the varying flavonoid content in different parts of *A. argyi*, we obtained the transcriptomes of four different tissue samples using next-generation sequencing. Prior to *de novo* assembly, we pre-processed a total of 448,665,104 reads generated from 12 paired-end libraries, which had a combined length of 67.7 Gb. After DynamicTrim and LengthSort filtering, the average length and ratio of trimmed to raw reads were 129.91 bp and 82.31%, respectively, resulting in a total of 429,154,582 clean reads with a total length of 55.7 Gb. These reads were assembled into 110,940 transcripts, of which 41,398 were selected as representative transcripts (unigenes) with an average length of 1,258 bp and an average mapping rate of 95.24%.

The highest number of assembled transcripts was found in the range of 500–1,000 bp for both the total (47,453) and representative (21,417) transcripts, and this number decreased as the length of the assembled transcripts increased (**Supplementary Table S1**). Approximately 71.63% of the unigenes (29,654) were successfully annotated in two public databases: NCBI-NR (Viridiplantae DB) and InterProScan. A summary of the RNA-seq data processing performed in this study is presented in **Table 1**.

**Table 1:** Summary statistics of RNA-seq, *de-novo* assembly, and annotation.

RNA-seq						
Data	Number of reads	Total length (bp)				
Raw data	448,665,104	67,748,430,704				
Trimmed data	429,154,582	55,751,105,490				
*De novo* assembly						
Data	Number of transcripts	Length (bp)				
		Total	Minimum	Maximum	Average	N50
Total transcripts	110,940	154,306,644	500	15,655	1,390	1,695
Representative transcripts	41,398	52,119,131	500	15,655	1,258	1,550
Annotation						
Data	NR-viridiplantae (rate)		InterProScan (rate)		Total annotation (rate)	
Total transcripts	87,657 (79.01%)		64,170 (57.84%)		87,958 (79.28%)	
Representative transcripts	29,511 (71.29%)		21,921 (52.95%)		29,654 (71.63%)	

### 3.4 Analysis of differentially expressed genes among different tissues of *A argyi*

Considering the differences in flavonoid content and anatomical characteristics among the four tissue samples, we examined two comparison groups: young leaves vs. old leaves, and trichomes vs. stems, to select differentially expressed genes (DEGs). A total of 3,493 (2,064 upregulated and 1,429 downregulated) and 5,157 DEGs (2,335 upregulated and 2,822 downregulated) were identified in young leaves vs old leaves, and trichomes vs stems, respectively (**Supplementary Figure S2**). The two sets of DEGs were functionally categorized using KEGG pathways. For both sets, the seven major classifications were “carbohydrate metabolism,” “signal transduction,” “biosynthesis of other secondary metabolites,” “environmental adaptation,” “lipid metabolism,” “amino acid metabolism,” and “metabolism of terpenoids and polyketides” (**Supplementary Figure S3A**). As our primary goal was related to secondary metabolites, we further focused on the “biosynthesis of other secondary metabolites” category. We confirmed that most genes related to phenylpropanoid, flavonoid, flavone, and flavonol biosynthesis were significantly and differentially expressed in both comparisons (**Supplementary Figure S3B**).

To examine the functional relationships between the DEGs and their associated biological processes, we mapped the DEGs to GO terms (**Supplementary Figure S4**). In the GO analysis of the “biological process” category, the most enriched term in the up-regulated DEGs of the young leaves vs old leaves group was “cell wall organization or biogenesis.” Conversely, the top enriched GO terms in the down-regulated DEGs of the young leaves vs old leaves group were strongly related to the response to various endogenous and external stimuli, such as “response to chemical” and “response to external biotic stimulus” (**Supplementary Figure S4A**). For the GO analysis of the “biological process” category of trichome vs stem DEGs, the most enriched term in the up-regulation was “secondary metabolic process” (**Supplementary Figure S4B**). Its relevant terms derived from the general phenylpropanoid pathway were particularly enriched, including “flavonoid biosynthetic process,” “phenylpropanoid biosynthetic process,” and “anthocyanin-containing compound biosynthetic process.”

### 3.5 Identification of candidate genes involved in eupatilin and jaceosidin biosynthesis

**Figure 4:**
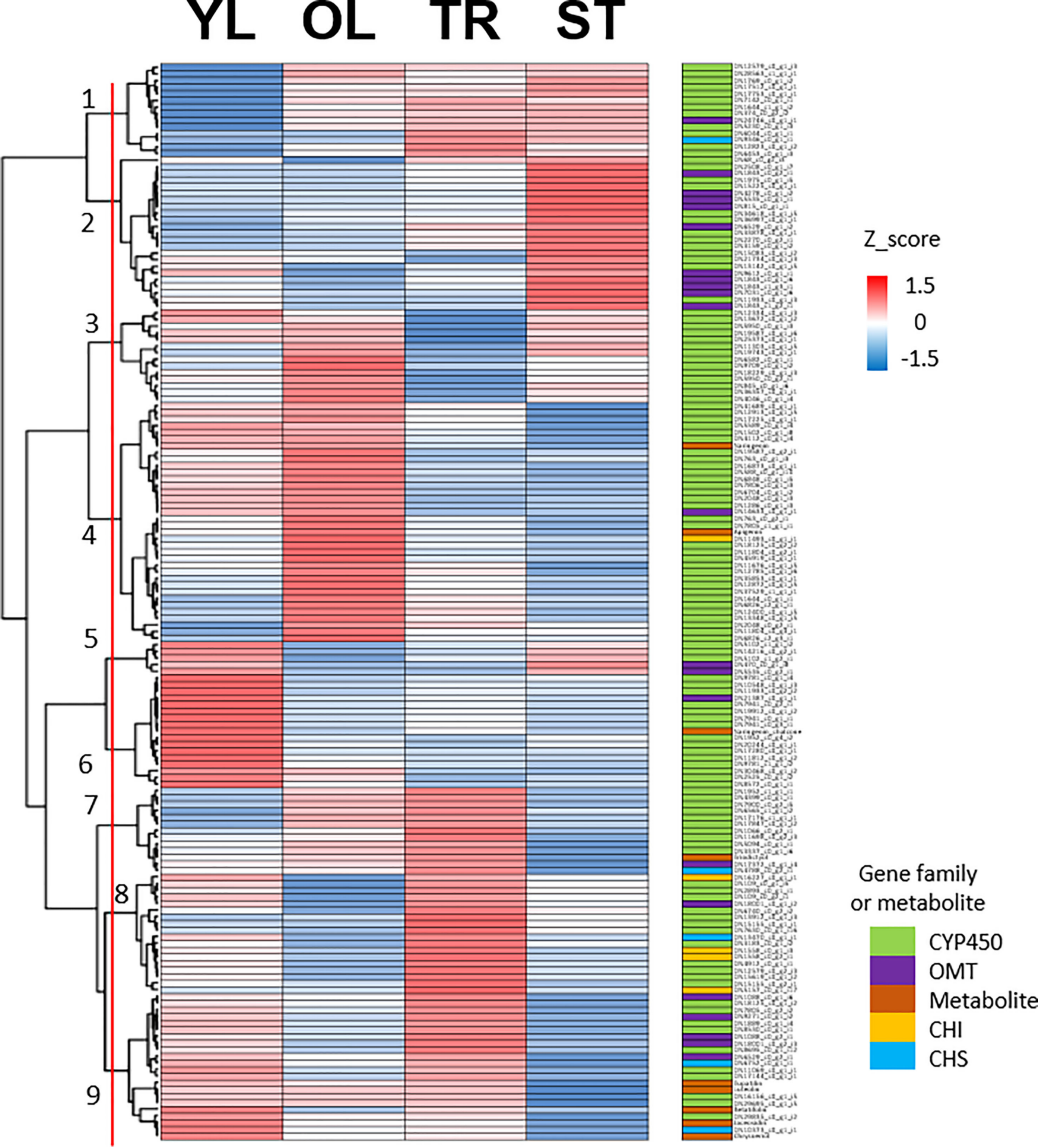
Clustering analysis of 153 flavonoid-related genes and 9 metabolites. Hierarchical clustering heat map of 153 flavonoid-related gene expression and content of 9 metabolites from four different tissues based on the z-score of either gene: Log_2_(normalized count+1) or metabolite: Log_2_(amount of metabolite+1). Color classification for gene family and metabolite is as follows, CYP450; Cytochrome P450 (light green), OMT; *O*-methyltransferase (violet), Metabolite includes naringenin, chalcone, naringenin, apigenin, eriodictyol, luteolin, chrysoeriol, batatifolin, eupatilin and jaceosidin (amber), CHI; Chalcone-flavanone isomerase (yellow), CHS; Chalcone synthase (cyan). YL represents young leaves, OL represents old leaves, TR represents trichomes, and ST represents trichome-removed stem.

To identify putative key genes encoding enzymes for eupatilin and jaceosidin production in *A. argyi*, we first identified flavonoid-related genes among the DEGs. Based on functional annotation, we identified 153 flavonoid pathway-related genes among the 7,265 DEGs. These 153 genes were divided into four gene families:121 genes for Cytochrome P450 (*CYP450*), 22 genes for O-methyltransferases (*OMT*), five genes for chalcone isomerase (*CHI*), and five genes for chalcone synthase (*CHS*). The 153 flavonoid-related genes and nine flavonoids analyzed in this study were subjected to hierarchical clustering analysis based on Z-scores across the four different tissues, resulting in their classification into nine clusters (**Figure 4**). All five flavonoids (eupatilin, jaceosidin, and their putative intermediates luteolin, chrysoeriol, and batatifolin) were grouped into ‘cluster 9’ with 17 flavonoid-related genes, including 10 *CYP450*, 5 *OMT*, and 2 *CHS* genes (**Figure 4**, **Table 2**).

**Table 2:** Summary of clustering analysis results divided into 9 clusters.

	Total	C1	C2	C3	C4	C5	C6	C7	C8	C9
CYP450	121	12	13	14	32	3	15	10	12	10
OMT	22	1	10	0	1	2	1	1	1	5
Metabolite	9	0	0	0	2	0	1	1	0	5
CHI	5	0	0	0	1	0	0	0	4	0
CHS	5	1	0	0	0	0	0	1	1	2
Sum	162	14	23	14	36	5	17	13	18	22

**Figure 5:**
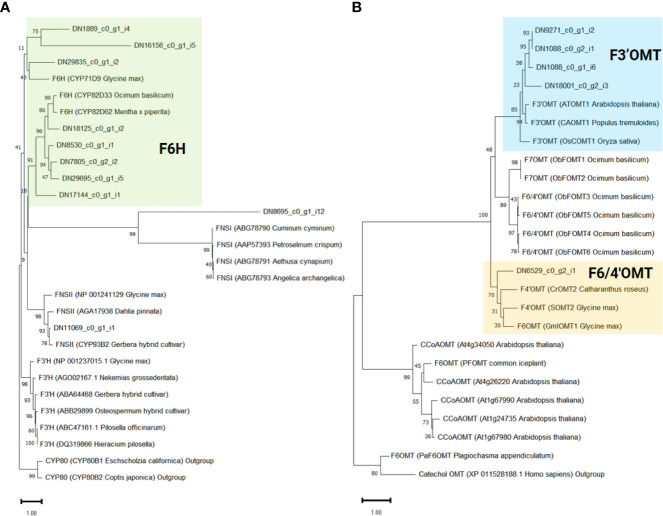
Phylogenetic analysis of flavonoid-related genes identified in ‘cluster 9’. The support values were obtained after 1,000 tests and indicated near the nodes. Protein sequences of previously known **(A)** Cytochrome P450 and 2-oxoglutarate-dependent dioxygenase and **(B)** *O*-methyltransferase genes were obtained from the NCBI database. The protein ID and the derived species information are enclosed in parentheses.

To infer the potential functions of the 10 genes annotated as *CYP450* in ‘cluster 9’, and to pinpoint flavone-6-hydorxylase (*F6H*) genes, we conducted a phylogenetic analysis. For comparison, we included previously characterized flavonoid biosynthetic CYP450 (F3’H, F6H, and FNSII), 2-oxoglutarate-dependent dioxygenase (FNSI), and irrelevant CYP450 family member, CYP80 as outgroup (**Figure 5A**). As a result, eight genes (DN1889_c0_g1_i4, DN16156_c0_g1_i5, DN29835_c0_g1_i2, DN18125_c0_g1_i2, DN7805_c0_g2_i2, DN8530_c0_g1_i1, DN17144_c0_g1_i1, and DN29695_c0_g1_i5) were classified as *F6H* genes required for the transition from chrysoeriol to batatifolin. Two other genes, DN8695_c0_g1_i12 and DN11069_c0_g1_i1, grouped with *FNS I* and *FNS II*, respectively, to convert naringenin to apigenin and/or eriodictyol to luteolin.

In our proposed pathway, *OMTs* are the key enzymes for the biosynthesis of eupatilin and jaceosidin. *F3’OMT* is required for the conversion of luteolin to chrysoeriol, and *F4’OMT*, *F6OMT*, or *F6/4’OMT* are required for the further *O*-methylation of batatifolin towards eupatilin and jaceosidin production (**Figure 3C**). To reveal which *OMTs* found in ‘cluster 9’ might function as *F3’OMT*, *F4’OMT*, *F6OMT*, or *F6/4’OMT*, phylogenetic analysis was performed with known *OMTs* (*F3’OMT*, *F4’OMT*, *F6OMT*, *F6/4’OMT*, and *F7OMT*). As a result, four *OMT* genes (DN1088_c0_g1_i6, DN1088_c0_g2_i1, DN9271_c0_g1_i2, and DN18001_c0_g2_i3) were classified as *F3’OMT*, and one gene (DN6529_c0_g2_i1) was classified as *F4’OMT* and/or *F6OMT* (**Figure 5B**).

**Figure 6:**
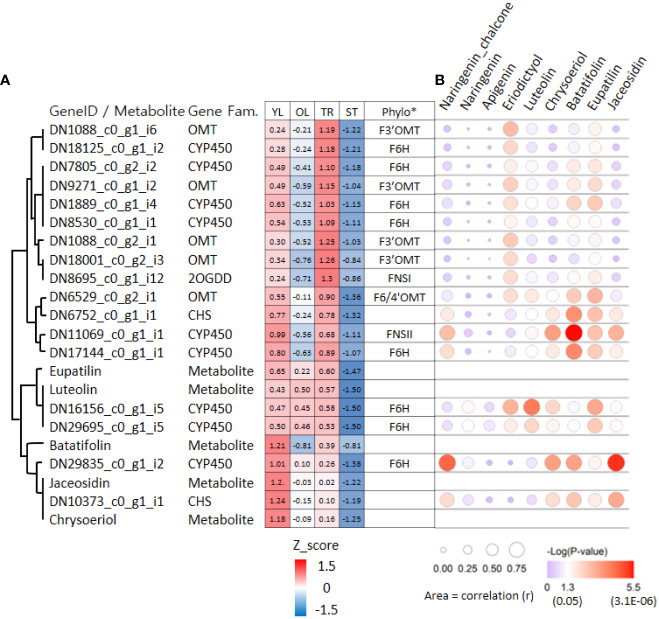
Hierarchical clustering heat map and bubble plots of putative key genes for jaceosidin and eupatilin biosynthesis. **(A)** Hierarchical clustering heat map of 17 genes and 5 metabolites derived from ‘cluster 9’ of **Figure 4**. Phylo* indicates putative functions of genes inferred through phylogenetic tree analysis (**Figure 5A**, 5B). Z-score calculated based on Log_2_(normalized count +1) or Log_2_(amount of metabolite+1). **(B)** Bubble plots showing an association between gene and 9 metabolites derived from Weighted gene co-expression network analysis (WGCNA). YL represents young leaves, OL represents old leaves, TR represents trichomes, and ST represents trichome-removed stem. The bubble plot shows the strength of the correlation (r) between two variables, represented by the size of each bubble. Larger bubbles indicate stronger correlations. The color of each bubble indicates the associated p-value.

To further investigate the association between genes and metabolites in ‘cluster 9’, we used the correlation values among 7,265 DEGs and metabolites obtained from weighted gene co-expression network analysis (WGCNA). Of the 17 genes in ‘cluster 9’, 16 (except DN18001_c0_g2_i3) were significantly associated with at least one of the five metabolites involved in the proposed pathway from luteolin to eupatilin and jaceosidin production (**Figure 6**). On average, these 17 genes were significantly associated with 2.6 metabolites. Two CYP450 genes (DN11069_c0_g1_i1 and DN17144_c0_g1_i1) were significantly associated with all five metabolites, whereas four genes (CHS: DN6752_c0_g1_i1, CYP450: DN29695_c0_g1_i5, CYP450: DN29835_c0_g1_i2, and CHS: DN10373_c0_g1_i1) were significantly associated with all four metabolites.

## 4 Discussion

### 4.1 Characteristics of trichomes in *A argyi* and their effect on metabolite content differences

In plants, trichomes can be divided into glandular and non-glandular types based on the presence or absence of glandular heads (Werker, 2000). Glandular trichomes are responsible for the synthesis, storage, and secretion of phytochemicals, and their distribution and density are linked to phytochemical production in various plants (Glas et al., 2012; Tissier, 2012; Cui et al., 2022). In contrast, non-glandular trichomes lack secretory mechanisms and mainly function in physical defense (Yamasaki and Murakami, 2014; Xiao et al., 2016).

Our measurements revealed that the eupatilin and jaceosidin contents were considerably higher in trichomes than in trichome-removed stems, indicating that these phytochemicals mainly accumulated in the trichomes of *A. argyi*. Additionally, eupatilin and jaceosidin were more abundant in young than in old leaves, likely owing to the higher density of glandular trichomes in developing young leaves (**Figures 2**, **3**). This interpretation is supported by a previous study showing that glandular trichomes are major accumulation sites for flavonoids in *A. argyi* (Cui et al., 2022).

### 4.2 Proposed eupatilin and jaceosidin biosynthesis pathway and candidate genes

Despite the medicinal importance of eupatilin and jaceosidin, their biosynthetic pathways have not yet been clearly elucidated in *A. argyi*. Determining the biosynthetic pathways of these natural products is challenging. However, insights into the necessary biochemical and enzymatic reactions can be gained from the chemical structures of the compounds of interest in their biosynthetic grids (Berim et al., 2012). For the production of eupatilin and jaceosidin, at least two or one step(s) of hydroxylation, respectively, and two additional steps of *O*-methylation are required, starting from the basic flavone skeletons, apigenin, or luteolin. Recently, a biosynthetic pathway starting from apigenin was proposed, using scutellarein and hispidulin as intermediates (Miao et al., 2022). However, the biosynthesis of hispidulin from scutellarein might be biochemically unfavorable because 6-*O*-methylation hardly occurs in the non-methoxylated flavone scutellarein (Berim et al., 2012). We propose an alternative biosynthetic route that utilizes chrysoeriol and batatifolin as intermediates (**Figure 3C**) and excludes biochemically unfavorable reactions. Supporting this proposed pathway, luteolin has been found to be an active substrate for F3´OMT producing chrysoeriol in rice during the biosynthesis of another polymethoxy flavone, tricin (Lam et al., 2021). For the production of eupatilin and jaceosidin, 6-*O*-methylation catalyzed by F6OMT or bifunctional F6/4´OMT is also indispensable. Thus, chrysoeriol must be further converted into 6-hydroxy chrysoeriol (batatifolin) *via* F6H, which provides a methyl group acceptor for -OH at the C6 position (**Figure 3C**). While proposing this alternative biosynthetic pathway, further loss-of-function study *in-planta* would be necessary to fully elucidate and confirm the biosynthetic pathway for these polymethoxy flavones.

According to the proposed pathways, chrysoeriol, batatifolin, jaceosidin, and eupatilin were observed to have relatively high levels of content in the young leaves, where the density of trichomes was higher than in the old leaves (**Figure 3C**). Moreover, the content of these substances was significantly higher in the trichomes when compared to the stem without trichomes (**Figure 3C**). These observations suggest that the biosynthesis or accumulation of these substances is specifically localized in the trichomes, and genes that are highly expressed in the trichomes or young leaves in contrast to the stem without trichomes or old leaves, might play a crucial role in this process. The co-expression of genes involved in the same metabolic pathway is often observed during biosynthetic processes (Saito et al., 2008; Nett et al., 2018), and such associations can be identified using transcriptome and metabolite data (Lau and Sattely, 2015). In our study, we used this approach for association analysis, which resulted in the identification of candidate genes (**Figure 4**). Further, phylogenetic tree analysis of genes with known functions (**Figure 5**) allowed us to identify the key genes necessary for eupatilin and jaceosidin biosynthesis in *A. argyi*. Identifying biosynthetic genes leads to a better understanding of plant biochemistry and enables the mass production of valuable phytochemicals through crop improvement and synthetic biology (Nett et al., 2018). Researchers have successfully co-expressed identified genes in alternative plant hosts (Lau and Sattely, 2015; Nett et al., 2020) and strains (Lau and Sattely, 2015; Nett et al., 2020) for the mass production of natural products. In particular, the biosynthesis of artemisinic acid, a precursor of artemisinin identified in *Artemisia annua*, was successfully performed in this strain (Paddon et al., 2013).

## 5 Conclusion

*Artemisia argyi* is a medicinal perennial plant with historical usage that exhibits pharmacological effects such as anti-inflammatory, anticancer, and antimicrobial activities, which are attributed to various secondary metabolites. In this study, we analyzed the flavonoid content of four different tissues of *A. argyi* and proposed the biosynthetic pathways of eupatilin and jaceosidin based on metabolite analysis and chemical structures. Through the examination of the chemical structures of the analyzed metabolites, we were able to identify the eupatilin and jaceosidin biosynthetic pathways. We performed *de-novo* assembly of transcriptome data from these tissues, resulting in 41,398 unigenes. Through a variety of analyses including DEG analysis, KEGG and GO term enrichment analysis, clustering, phylogenetic tree construction, and WGCNA, we identified candidate genes involved in the biosynthesis of eupatilin and jaceosidin. Our work serves as a basis for the development of synthetic biological systems for the mass production of these compounds in the future.

## Data availability statement

The data presented in the study are deposited in the NCBI repository, accession number PRJNA945494.

## Author contributions

SL, HW, and SB collected and analyzed the data, and wrote the manuscript. YP, SK, HK, JC, and H-YK collected data. JL and JJ designed the study, analyzed the data and wrote the manuscript. All authors contributed to the article and approved the submitted version.
