# Supplementary material for: Integrative analysis of metabolite and transcriptome reveals biosynthetic pathway and candidate genes for eupatilin and jaceosidin biosynthesis in Artemisia argyi
Source: Front Plant Sci. 2023 Apr 25;14:1186023. doi: 10.3389/fpls.2023.1186023 (PMC10166882; doi:10.3389/fpls.2023.1186023)
Supplement: Supplementary file 1 [file Presentation_1.pptx]

## Slide 1
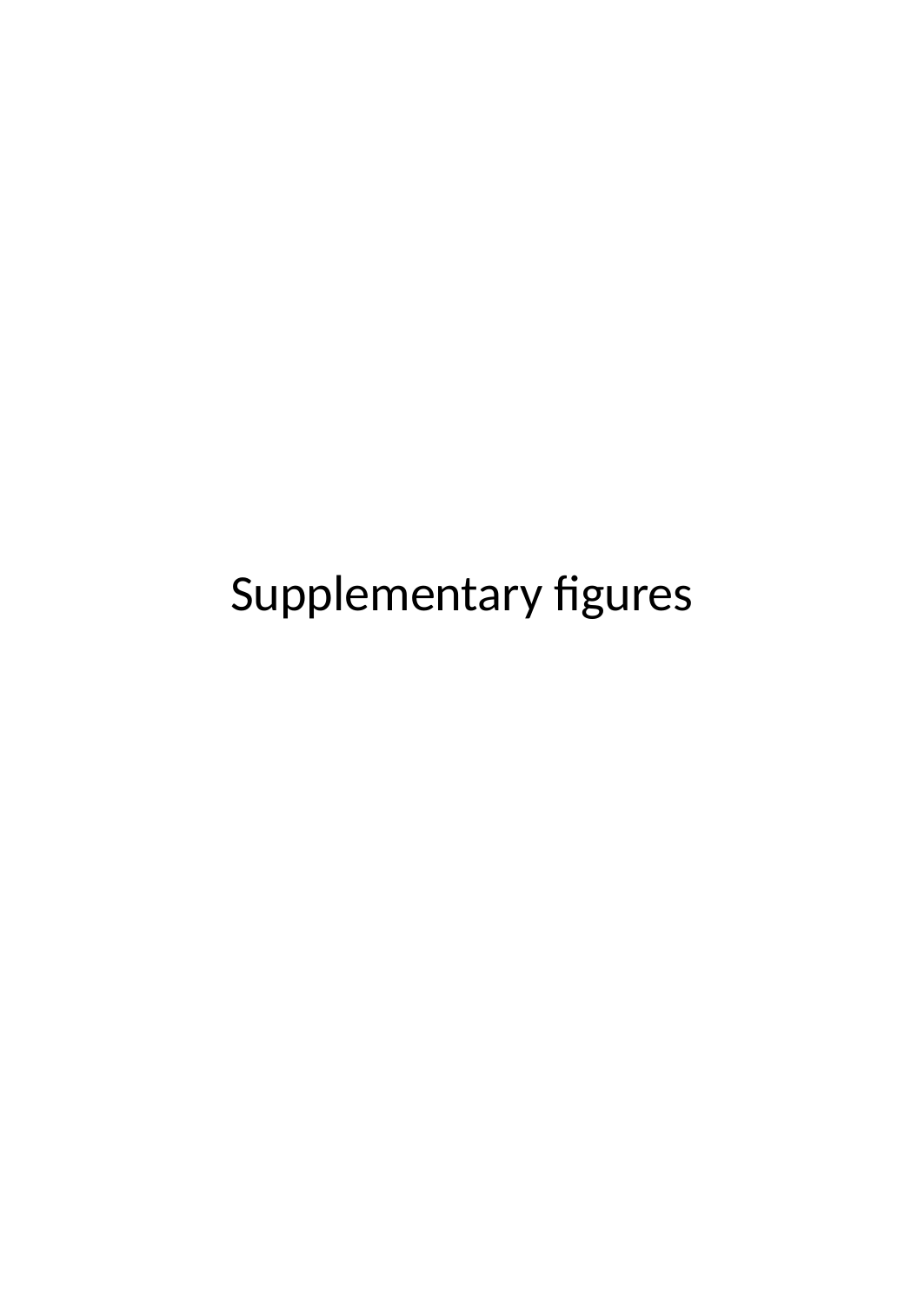

Supplementary figures

## Slide 2
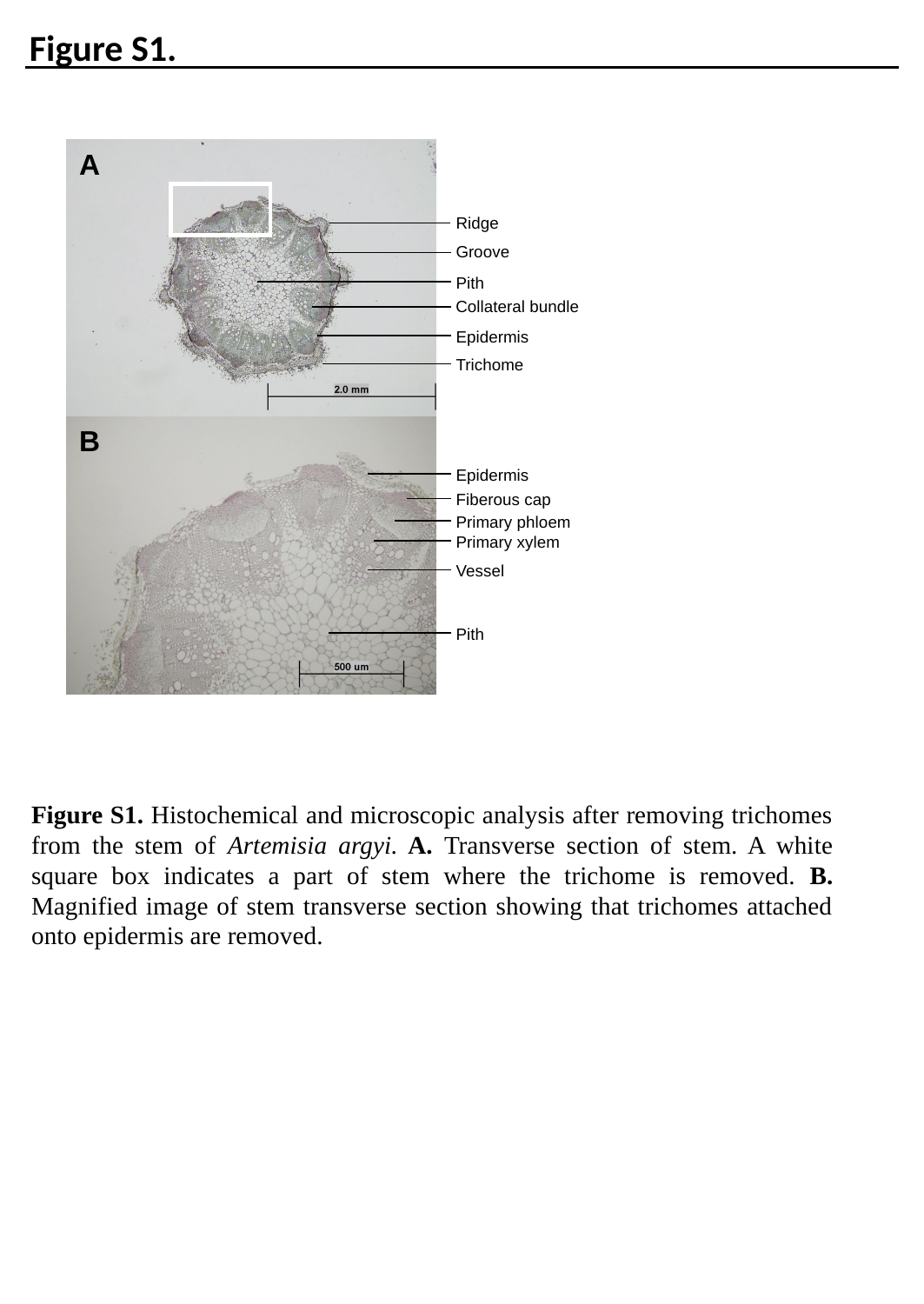

Figure S1.
A
Ridge
Groove
Pith
Collateral bundle
Epidermis
Trichome
B
Epidermis
Fiberous cap
Primary phloem
Primary xylem
Vessel
Pith
Figure S1. Histochemical and microscopic analysis after removing trichomes from the stem of Artemisia argyi. A. Transverse section of stem. A white square box indicates a part of stem where the trichome is removed. B. Magnified image of stem transverse section showing that trichomes attached onto epidermis are removed.

## Slide 3
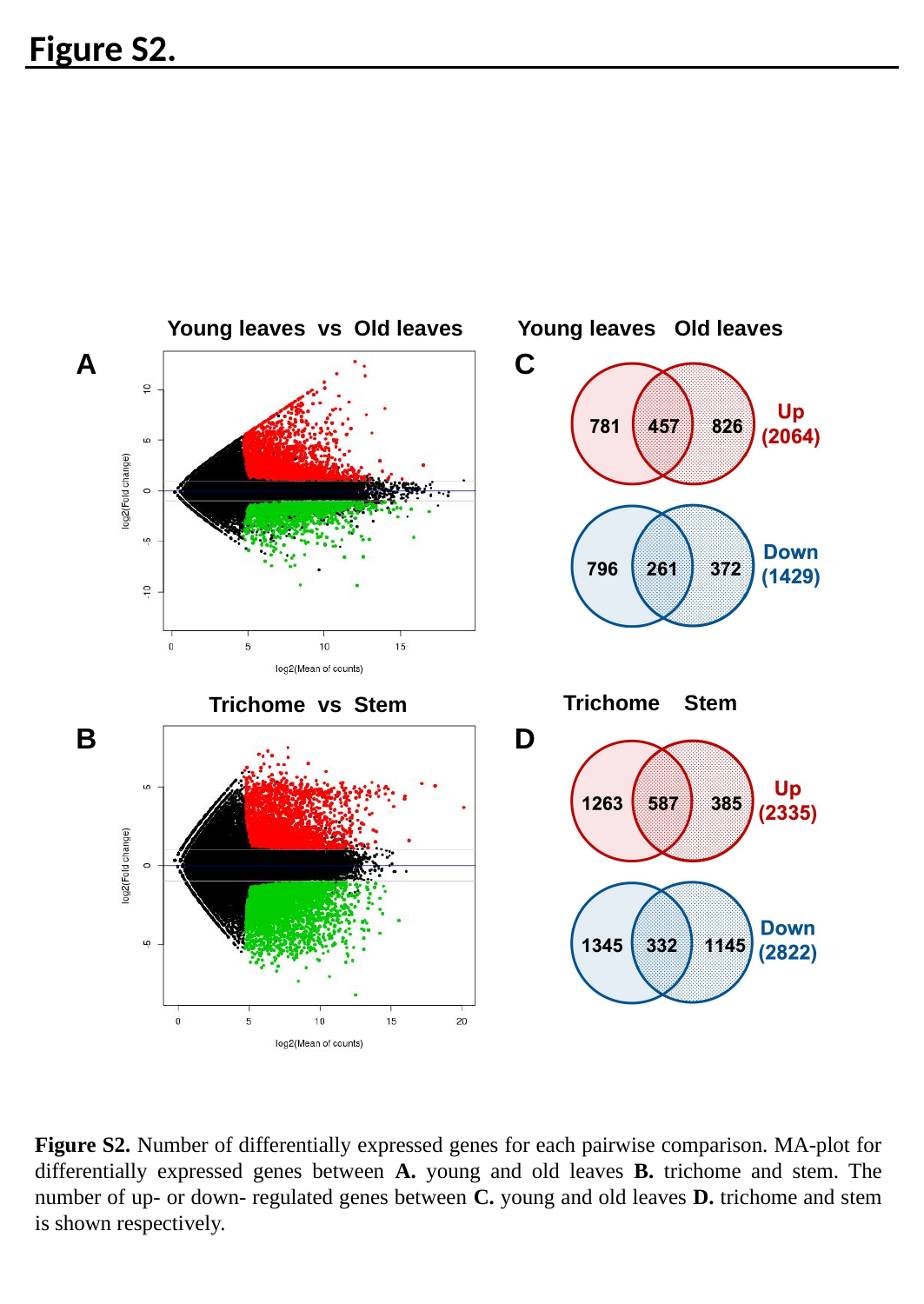

Figure S2.
A
C
B
D
Young leaves vs Old leaves
Young leaves Old leaves
Trichome Stem
Trichome vs Stem
Figure S2. Number of differentially expressed genes for each pairwise comparison. MA-plot for differentially expressed genes between A. young and old leaves B. trichome and stem. The number of up- or down- regulated genes between C. young and old leaves D. trichome and stem is shown respectively.

## Slide 4
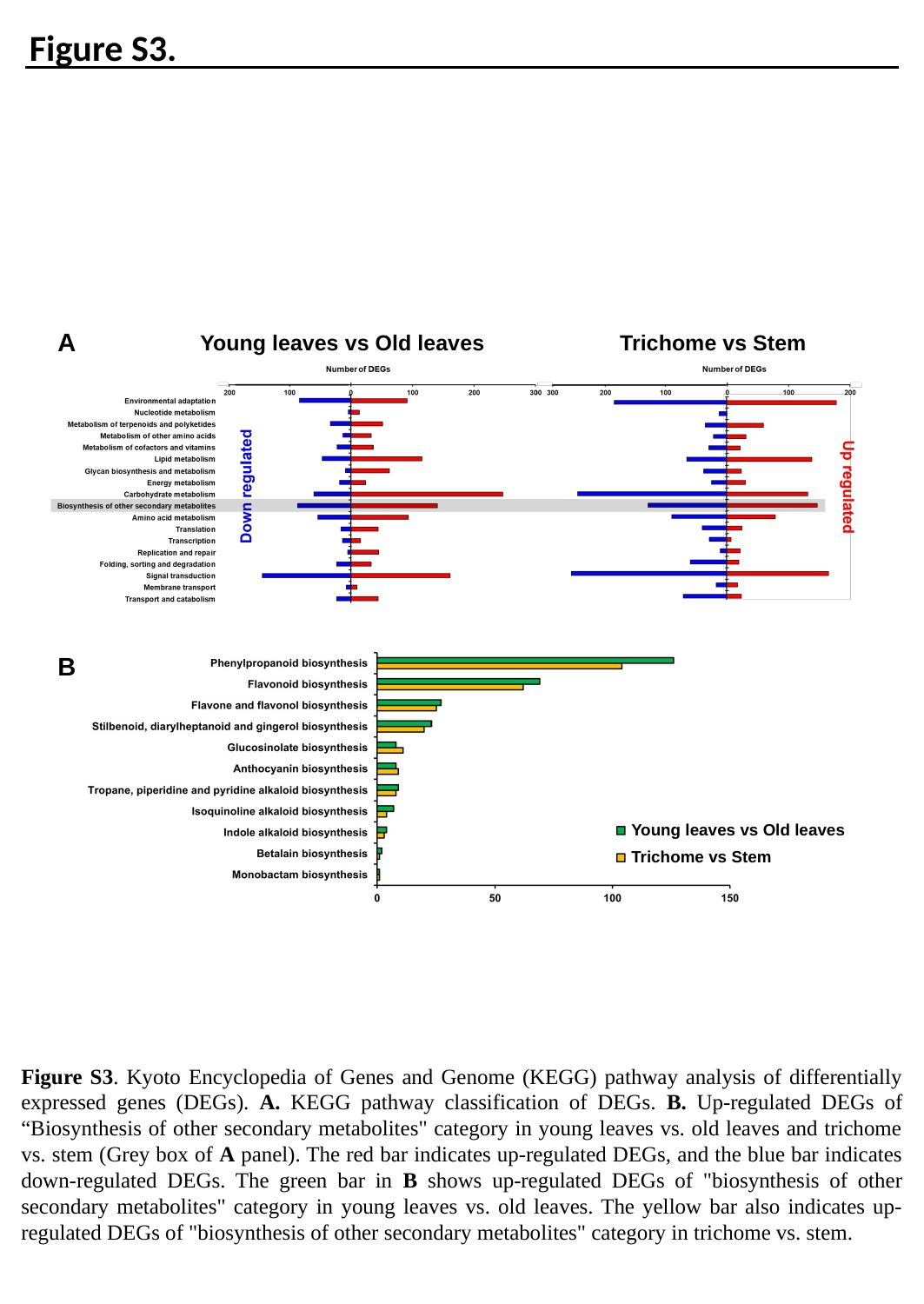

Figure S3.
A
B
Young leaves vs Old leaves
Trichome vs Stem
Young leaves vs Old leaves
Trichome vs Stem
Figure S3. Kyoto Encyclopedia of Genes and Genome (KEGG) pathway analysis of differentially expressed genes (DEGs). A. KEGG pathway classification of DEGs. B. Up-regulated DEGs of “Biosynthesis of other secondary metabolites" category in young leaves vs. old leaves and trichome vs. stem (Grey box of A panel). The red bar indicates up-regulated DEGs, and the blue bar indicates down-regulated DEGs. The green bar in B shows up-regulated DEGs of "biosynthesis of other secondary metabolites" category in young leaves vs. old leaves. The yellow bar also indicates up-regulated DEGs of "biosynthesis of other secondary metabolites" category in trichome vs. stem.

## Slide 5
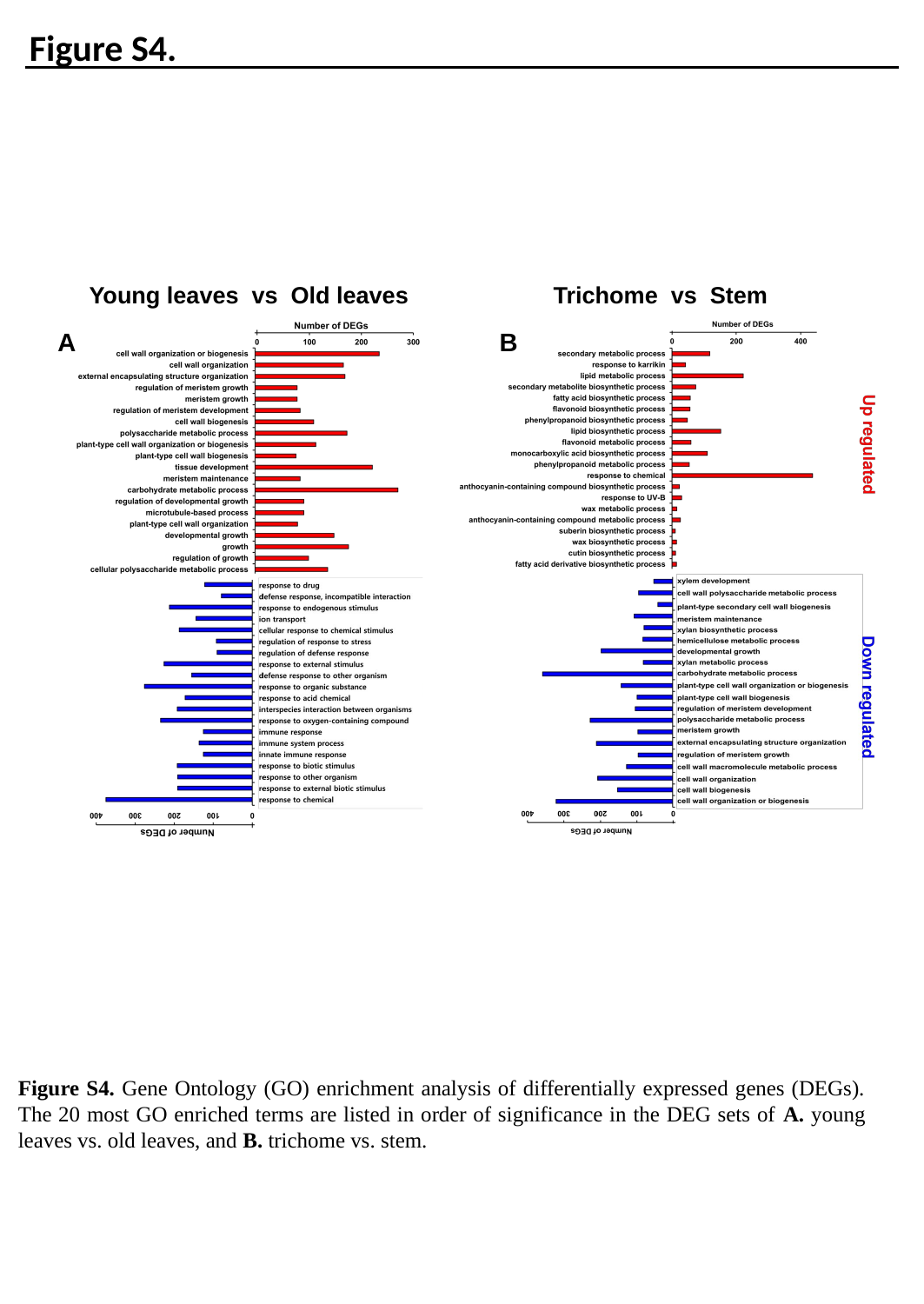

Figure S4.
Trichome vs Stem
Young leaves vs Old leaves
A
B
Figure S4. Gene Ontology (GO) enrichment analysis of differentially expressed genes (DEGs). The 20 most GO enriched terms are listed in order of significance in the DEG sets of A. young leaves vs. old leaves, and B. trichome vs. stem.

## Slide 6
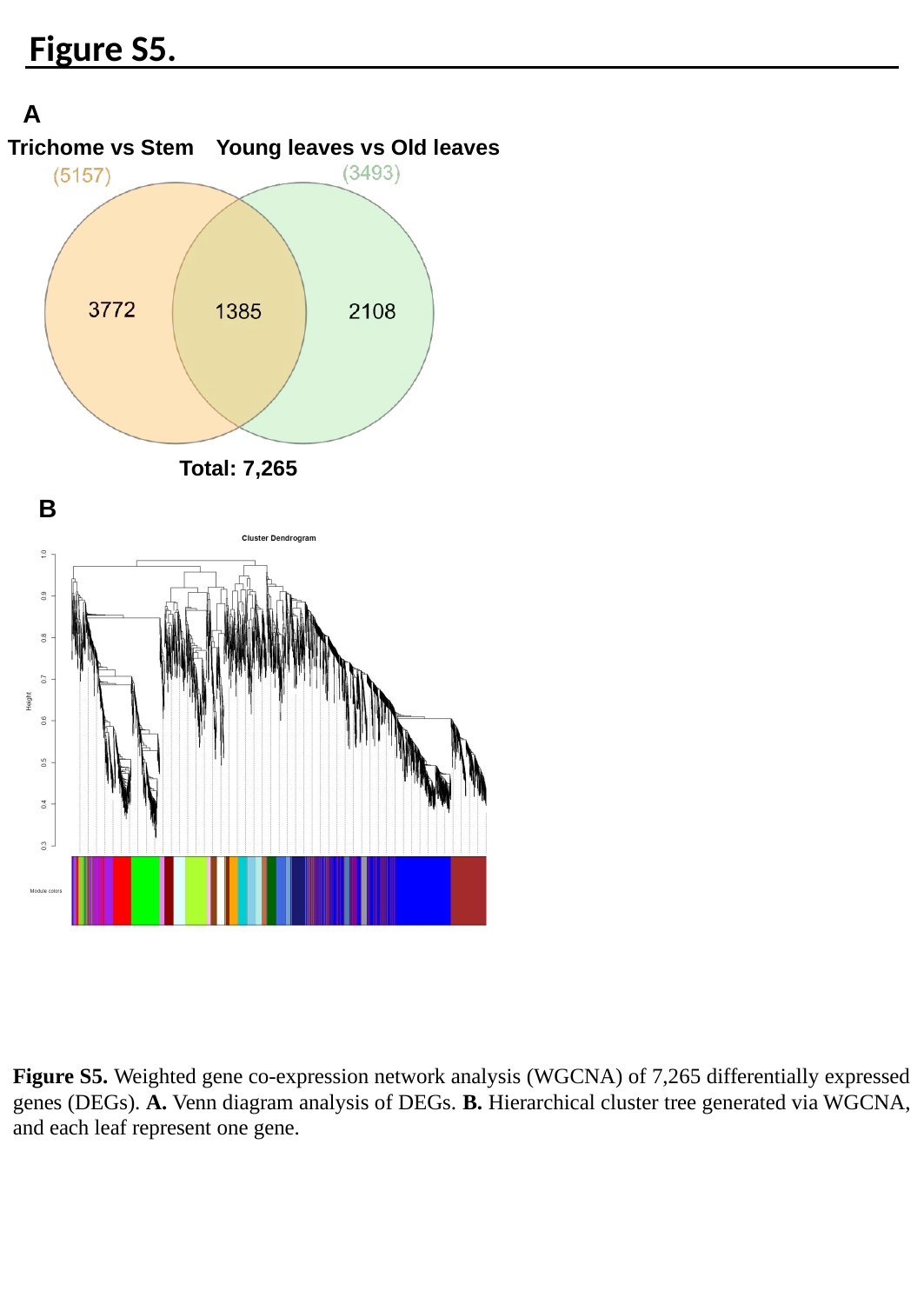

Figure S5.
A
Young leaves vs Old leaves
Trichome vs Stem
Total: 7,265
B
Figure S5. Weighted gene co-expression network analysis (WGCNA) of 7,265 differentially expressed genes (DEGs). A. Venn diagram analysis of DEGs. B. Hierarchical cluster tree generated via WGCNA, and each leaf represent one gene.

## Slide 7
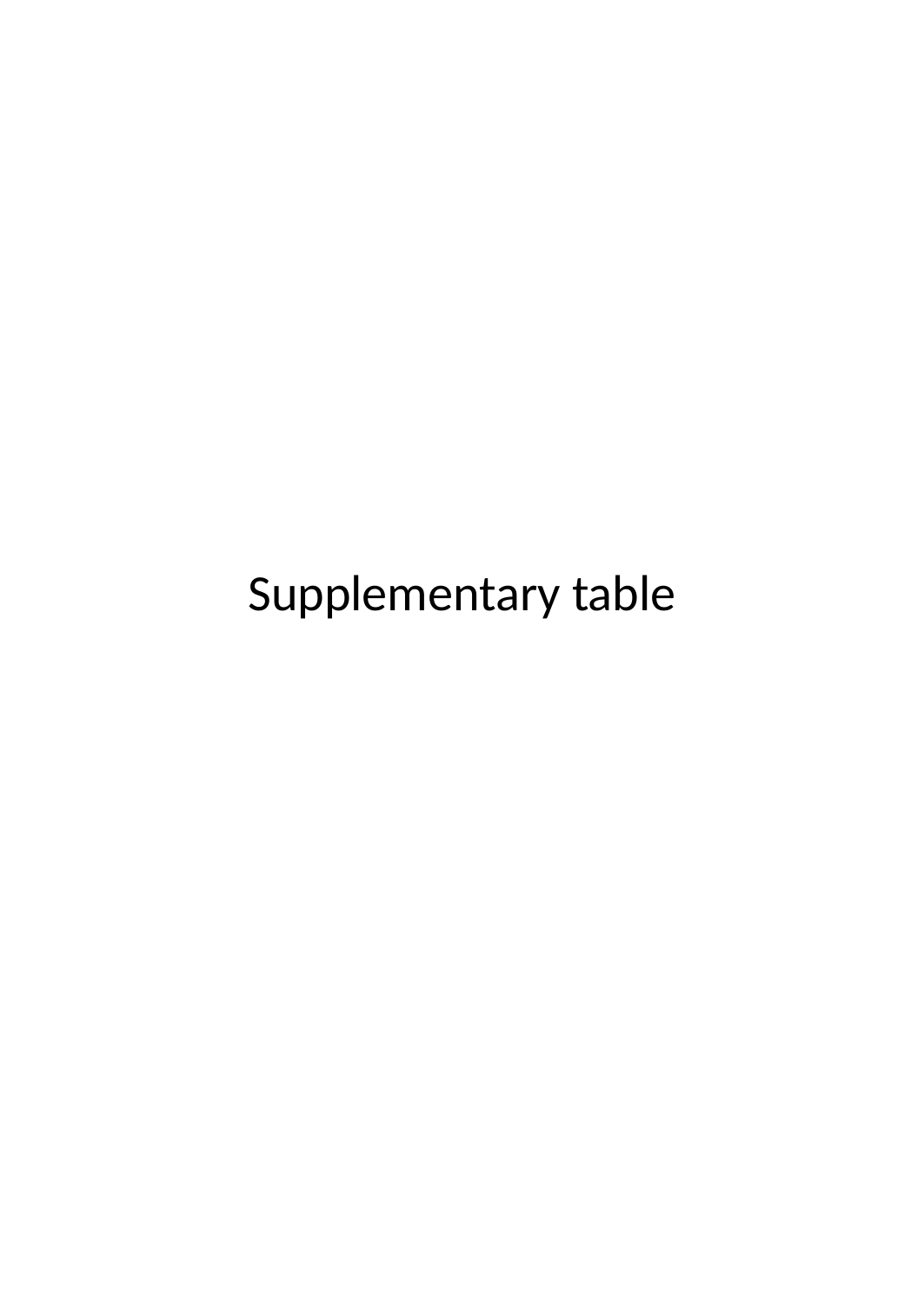

Supplementary table

## Slide 8
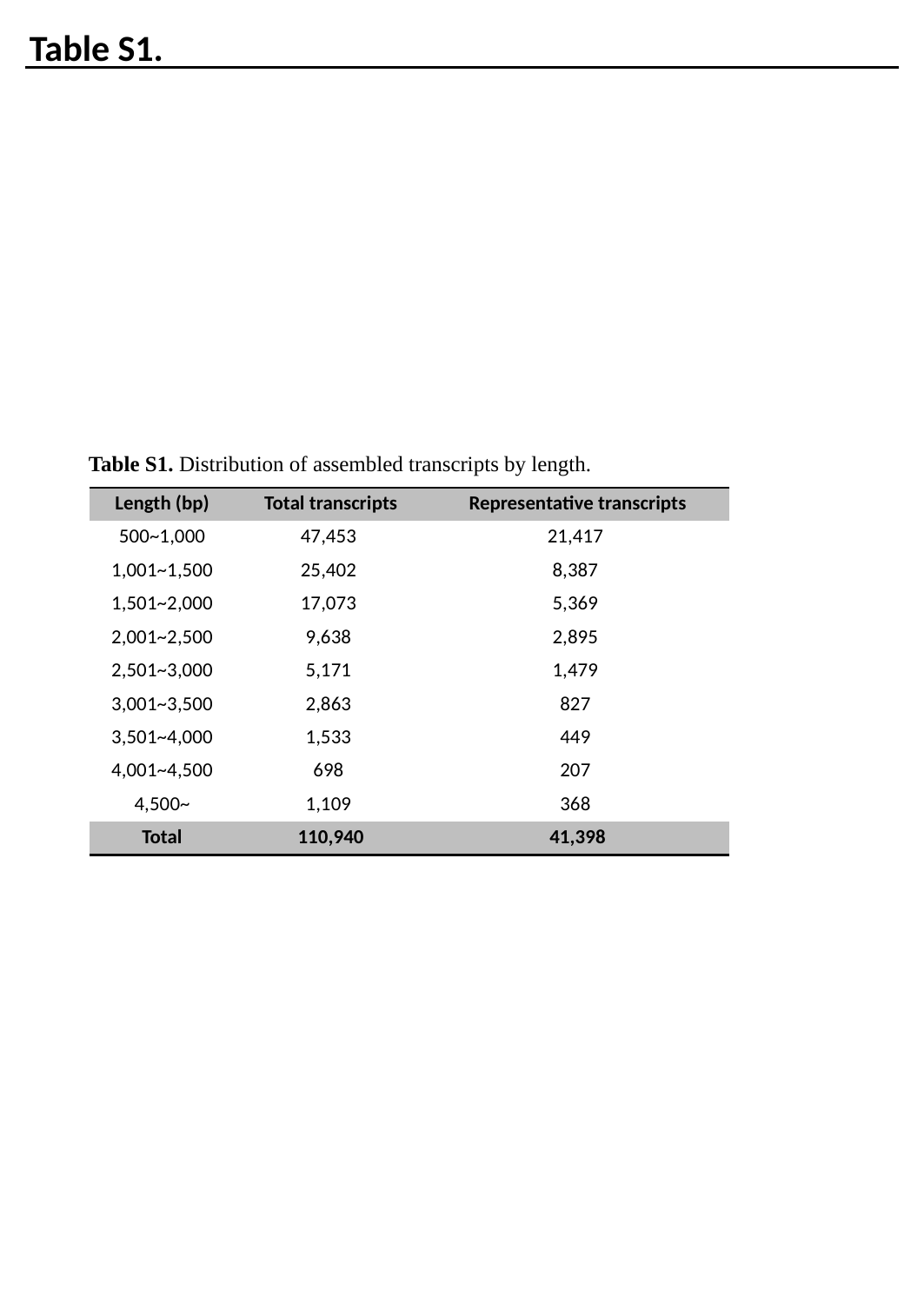

Table S1.
Table S1. Distribution of assembled transcripts by length.
| Length (bp) | Total transcripts | Representative transcripts |
| --- | --- | --- |
| 500~1,000 | 47,453 | 21,417 |
| 1,001~1,500 | 25,402 | 8,387 |
| 1,501~2,000 | 17,073 | 5,369 |
| 2,001~2,500 | 9,638 | 2,895 |
| 2,501~3,000 | 5,171 | 1,479 |
| 3,001~3,500 | 2,863 | 827 |
| 3,501~4,000 | 1,533 | 449 |
| 4,001~4,500 | 698 | 207 |
| 4,500~ | 1,109 | 368 |
| Total | 110,940 | 41,398 |
